# Networks of Negativity: Gaining Attention Through Cyberbullying

**DOI:** 10.3390/ijerph21121699

**Published:** 2024-12-20

**Authors:** Diane Felmlee, Sara Francisco, Melissa Hardy

**Affiliations:** 1 Department of Sociology and Criminology, The Pennsylvania State University, University Park, PA 16802, USA; melissa.hardy@psu.edu; 2 Department of Sociology, Grinnell College, Grinnell, IA 50112, USA; franciscos@grinnell.edu

**Keywords:** cyberbullying, social networks, Twitter, sexism, racism

## Abstract

Cyberbullying entails multiple, problematic consequences for its victims. However, little is known about the factors that influence the dispersion of these damaging messages. Drawing on theories of social interaction, we argue that perpetrators disseminate messages containing sexist and racist slurs that reinforce stereotypical, social norms to enhance their online visibility. We collected cross-sectional and longitudinal samples of tweets (N = 539,558 and 248,497, respectively) that included either gender or Asian slurs. We find that tweets containing gender or Asian slurs that were more negative in sentiment had a significantly higher number of retweets than more positive tweets, thereby heightening a user’s online presence and the reach of their content. Two historical events unfolded during our study—the onset of COVID-19 and the murder of George Floyd by a police officer. Tweet negativity increased following both events. Our findings demonstrate the capacity of aggressive tweets to generate wide-ranging networks, a process which is exacerbated further by public crises. Results suggest that the practice of sending such messages is strategic. Perpetrators likely engage in cyberbullying, consciously or not, to increase their online attention. Moreover, this strategy appears to be successful.

## 1. Introduction

Internet communication platforms constitute increasingly important locations for conversations and, in some cases, substitute for face-to-face interaction. While these platforms may provide significant social and economic benefits, they can simultaneously incubate problematic and abusive activities. In particular, expanding reliance on social media communication amplifies the dissemination of offensive speech to audiences of like-minded as well as curious viewers. 

In today’s world, viewers routinely find aggressive, harmful messages on social media, with approximately three-quarters of American internet users (72%) having witnessed at least one form of cyberbullying [1], which refers to the application of technology to harm, threaten, target, or harass another person. Close to half a million sexist slurs occur daily on social media, all of which can spread widely [2]. Adolescents and children who receive these negative messages often experience a range of adverse psychological, emotional, and behavioral aftereffects [3,4,5]. Adult targets of cyberbullying also suffer multiple adverse consequences, such as anxiety, panic attacks, suicidal ideation, and depression [6], although adult cyberbullying receives much less attention in the literature. Furthermore, relatively little is known about the social factors that prompt adults to endorse and circulate damaging online messages that often attack individuals belonging to historically disadvantaged groups. In this project, we discuss social processes that may contribute to networks of cyberbullying on the social media platform Twitter (now known as X), focusing on posts that frequently draw from and further reinforce gender and race stereotypes.

We argue that two group processes derived from classical social interaction theories may contribute to electronic forms of aggression [7]. The first process is the establishment of “pecking orders,” or status hierarchies, and the second involves the reinforcement of social norms. Perpetrators can use social media to increase their virtual standing by sending messages that they expect will gain attention and be retweeted widely. Messages, or tweets, that are more likely to draw notice may be those reflecting norms that highlight social stereotypes and reinforce prejudice, such as those critical of women and people of color. Therefore, we anticipate that the digital script of many negative messages will echo traditional, harmful stereotypes. Given that forms of social media are increasingly influential in society and play a notable part in shaping politics [8,9,10,11], the dissemination of stereotypes in these venues represents an essential topic of study.

Our goal is to examine whether the negativity of tweets that include either sexist or racist slurs is associated with greater online attention in the form of retweets. In addition to studying retweets in a cross-sectional sample, we also follow a sample of users to see whether tweets with higher levels of negativity receive wider exposure over time through the accumulation of retweets. Finally, our data collection coincided with two significant U.S. events—the onset of the COVID-19 pandemic and the murder of George Floyd, a Black man, by a White police officer. Our analysis therefore controls for both events and investigates whether these socially meaningful and politically controversial events serve as potential triggers for exacerbating negative sentiment.

## 2. Theoretical Framework

Numerous studies document repeated instances of cyberbullying, online harassment, internet bullying, or online victimization, terms that are often used interchangeably. According to prior surveys, for example, 41–47% of Americans report experiencing online harassment [1,12]. The frequency of more severe forms of harassment have increased, with one-quarter of respondents recounting relatively extreme versions of harassment in 2021, such as physical threats, stalking, sustained harassment, and/or sexual harassment, as compared to 15% in 2014 [12].

Cyberbullying can be relentless, occur repeatedly at any time of the day or night, spread widely, and be anonymous, which can exacerbate harm to teens and children. One systematic review of the effects of cyberbullying on children and young adults, for example, found a strong negative link to mental health outcomes. Depression, anxiety, hostility/aggression, and suicidality are some of the most frequent mental health consequences, whereas problems with self-esteem and peer relationships were two common psychosocial outcomes [13]. Adults also suffer from online abuse. According to a systematic review [6], 42 of 43 studies found evidence that adult victims of cyber harassment and/or cyber stalking faced harmful repercussions, such as depression, anxiety, suicidal ideation, and panic attacks.

### 2.1. Gender, Race, and Cyberbullying

Digital forms of bullying and harassment vary in frequency and consequences by gender. For example, close to half of women report being harassed online due to their gender, as compared to 18% of men [12]. Studies examining how cyberbullying and online harassment target and affect women reveal that negative messages aimed at women can reinforce traditional feminine stereotypes, including expectations of physical beauty, sexual “purity,” and temperaments that are soft, pleasant, or kind [2,14,15,16]. Additionally, online content may aim to demean women and can contain threats of sexual violence [16,17]. In a study by Vickery [18], many men were found to view these digital spaces as primarily masculine platforms. As women join these venues, a misogynist backlash may be present, allowing men to “reclaim” these spaces and contribute to the rise of online abuse and aggression toward women [14].

Cyberbullying also varies by race and ethnicity. Approximately 29% of individuals experiencing virtual bullying in 2020 attributed it to their race or ethnicity [12]. In the spring of 2020, moreover, racial harassment, antagonism, and cyber racism toward Asians increased greatly in response to the beginning of COVID-19 [19,20].

Prior research on discrimination toward Asian Americans often focused on the “model minority” stereotype, with White adults viewing Asian Americans as cold, yet competent [21]. However, the increase in Asian immigration beginning in the 1800s had promoted negative stereotypes and discriminatory laws. The expansion of Asian neighborhoods fueled their perceived threat to existing business and cultural practices. After COVID-19, Asian stereotypes shifted to the threat of the disease, invoking the historical roots of the “Yellow Peril”. In one 2020 poll, 49% of Americans believed the virus to be an agent of biological warfare intentionally created by China [22]. Moreover, Budhwani and Sun [23] reported nearly a ten-fold increase on Twitter in the use of stigmatizing terms such as “China virus.” Therefore, given the overlap of our study with the onset of COVID-19, we focus on anti-Asian tweets, rather than those targeting other race/ethnic groups.

### 2.2. Theories of Cyberbullying

Group processes leading to the development of social norms and status hierarchies are relevant to cyberbullying and aggression. According to classic, social interaction theories [7,24], norms and hierarchies represent fundamental developments that arise in systems of human relations. When people interact with others, they acquire and enforce social norms that encourage conformity to established social expectations and discourage resistance and rebellion. Moreover, an individual’s interactions result in the unfolding of status hierarchies in which certain individuals attain greater respect and esteem than others. We argue that these basic social processes contribute to the formation of cyberbullying.

Individuals who engage in aggression and bullying routinely compete for recognition, status, and popularity both online and face to face [25,26]. Perpetrators of harassing and aggressive messages are apt to be motivated to gain attention from others online to increase their standing in the form of a wider digital footprint. One approach used to obtain notice is through posting tweets that will be retweeted by others, subsequently spreading messages to a larger audience, and in some cases extending circulation far beyond the initial reach.

Online responses in the form of retweets do not always provide support for the content of the original message. Retweets can serve as endorsements from a supporter, documentation from a journalist, or condemnation from a dissenter. Regardless of an individual’s intent for reposting a message, the retweet expands the attention received by the tweet even if the additional attention is antagonistic rather than supportive.

Mixed evidence exists on whether positive or negative sentiment messages receive more retweets. Eye-tracking experiments reveal that social media posts containing positive images, rather than negative or no images, prompted increases in visual attention by participants as well as a greater intention to share a post [27]. According to an in-depth study of responses to news articles on the New York Times Web Site [28], positive articles enhance the chances that people share with others online. In contrast, messages on Twitter with negative content, such as adverse comments on political events, receive more attention than those with positive content [29,30,31,32].

### 2.3. Focus of Our Study

Existing research says little about any correlation between the negativity of Twitter posts containing sexist or racist slurs and the augmentation of potential audiences through retweets. We use theories of social interaction and empirical findings to motivate a study of this issue—the connection between tweets’ sexist/racial negativity and their diffusion. We anticipate that more negative sentiment will be associated with greater numbers of retweets both cross-sectionally and longitudinally. Based on our discussion above, we test the following hypotheses:

**H1.**  *Tweets with more negative sentiment will garner more retweets in cross-sectional samples of tweets.*

**H2.**  *Based on our longitudinal sample of users, tweets containing more negative sentiment will accumulate more retweets over time.*

In addition, social interactions occur within a larger context defined by current circumstances and ongoing events. Two crucial events intervened during our data collection. First, on 11 March 2020, COVID-19 was declared a pandemic by the World Health Organization, drastically changing everyday routines, reorganizing work and family life, limiting social contact and heightening a reliance on forms of digital communication. The second significant event was the murder of George Floyd in Minneapolis, MN on 25 May 2020 by a police officer, Derek Chauvin, leading to protests opposing police brutality and societal racism across the United States and internationally [33,34]. Both events reinforced race/ethnic social categories, although in different ways. And both fueled a heightened sense of threat and vulnerability. Therefore, we expect that:

**H3.**  *Both events will amplify the negative emotional content of Twitter posts.*

## 3. Methodology

We estimate cross-sectional regression models using data from stacked, daily, cross-sectional slices of tweets, which allow us to test whether more negative tweets tally more retweets. We also estimate longitudinal models on negative tweets from a subset of users. These models assess whether the negativity of their posts promotes sustained attention over time by accumulating more retweets as days pass.

### 3.1. Cross-Sectional Data

Over several months between 2019 and 2020, we collected two sets of tweets from the Twitter API via academic research developer accounts. We chose this period, because of increasing concern regarding the role of social media in the spread of racist and sexist content. We scraped Twitter and collected all publicly available tweets containing contentious terms related to gender (one set of tweets) and racial insults focusing on Asians (the second set of tweets). This research was conducted with the approval of the Pennsylvania State University Institutional Review Board (STUDY00004666), which waived the requirement for informed consent.

We searched for tweets that included one of four gender keywords (“b*tch,” “c*nt,” “sl*t,” and “wh*re”). These gendered slurs were chosen based upon their high rank within the top 20 most frequently used curse words on Twitter [35]. They also represent the most prevalent, derogatory feminine slurs in a sample of tweets [2]. Simultaneously, we gathered tweets that invoked misleading stereotypes linking COVID-19 to Asians. We chose to include the following terms related to anti-Asian racism and xenophobia mentioned by government officials and public rhetoric: “Chinese virus”, “ch*nk”, “hong kong flu”, “kung flu”, and “Asian” [36]. Although “Asian” itself is not a slur, given the widespread anti-Asian bullying that occurred during the pandemic using phrases such as “Asian virus” [37], we included the term in our initial analyses. The final dataset consists of 539,558 tweets. The frequencies of these keywords can be found in Table 1.

### 3.2. Longitudinal Data

For the longitudinal analyses, we randomly selected 94 users who included the term b*tch at least once and followed their tweets from 2 December 2019, until 11 June 2020. We also followed 297 users who tweeted at least one of the Asian terms over the same period. Our final sample for the longitudinal dataset includes 248,497 tweets from 391 distinct users. See Table 2 for the frequencies of these key words.

### 3.3. Measuring Sentiment

We apply a sentiment classifier specifically honed to identify cyberbullying on Twitter to assess the emotional content of the tweets in our samples [2]. This supervised classifier relies on a lexicon built upon tweet language and represents an ensemble of three popular, sentiment analysis approaches (i.e., VADER, “bing” and “afinn;” (https://github.com/cjhutto/vaderSentiment (accessed on 15 December 2024))) to compute a final score. This approach removes word order as well as extremely common words (i.e., “stop words”) so that the sentiment score of each tweet is determined by the sum of the scores of the individual words remaining in each tweet. The final sentiment score associated with each tweet ranges from extremely negative (−4) to extremely positive (+4).

The ensemble sentiment classifier performed well in comparison to scores on a test set of 400 tweets obtained from four, ethnically diverse, human coders, with overall F1 scores of 0.746 (micro) and 0.697 (macro). The F1 scores reflect a weighted harmonic mean of the precision and recall of the algorithm, with a minimum value of 0 and maximum of 1. These scores indicate an improvement over those obtained from any one of the more common classifiers (e.g., VADER) or from other combinations of them, which is one of the main reasons that we chose this customized classifier.

### 3.4. Multivariate Approach

We test our hypotheses using ordinary least squares (OLS) regression models that specify our key variables and controls and are estimated using a dataset that combines tweets containing gender and Asian insults. The dependent variable in our models is the number of retweets associated with each tweet (logged to adjust for skewness).

For the longitudinal analyses, we estimate fixed-effect, lagged, OLS regression models on the combined dataset of tweets with either gender and/or Asian keywords. We include controls for the individual users in the fixed-effect analyses as we follow users over time. In the first set of longitudinal analyses, our dependent variable is the number of retweets associated with each tweet (logged to adjust for skewness). The predictors include: (1) the sentiment score associated with the tweet (−4 to 4), (2) a binary variable signaling the start of the COVID-19 Pandemic, and (3) a binary variable indicating the date of George Floyd’s murder (included only in the longitudinal analyses due to missing cross-sectional data). Control variables include several metrics from Twitter that could influence the dependent variable, including the number of friends, followers, and likes. The variable, “friends,” is the number of individuals the user chooses to see updates about, whereas “followers” focuses on the number of individuals that follow the user. “Likes” indicate how many “likes” or “favorites” the message received, while the measure of “retweets” was a count of the times a particular tweet was reposted by other users. We control for these variables, because they may indicate the influence of certain users or accounts in the spread of messages and content [38,39,40]. We employ a natural log transformation of these four predictors because they are continuous and display exponential, highly skewed distributions and are better suited to a proportional difference interpretation of association. Additionally, we control for “tweet length,” a continuous variable of the number of characters in a message, because longer messages might contribute to inflated negative sentiment.

Next, we examine whether tweets became more negative in sentiment following the onset of COVID-19 and the killing of George Floyd using each tweet’s sentiment score as the dependent variable. We use the same predictors as in the previous longitudinal model but add the number of retweets as a predictor. We include these two event variables within our models for three reasons. First, the onset of the COVID-19 pandemic resulted in additional public health safety measures and practices including self-isolation and shelter-in-place ordinances, which contributed to an increased use in social media [41]. Second, Floyd’s death occurred during the height of the COVID-19 pandemic when engagement with social media reached unprecedented levels [42]. Last, research finds that the intersection of the COVID-19 pandemic and racial tensions within the United States are connected to problematic social media usage where individuals may become fatigued, and unable to process and consume the information they receive online [43,44]. Thus, we test whether users who tweet negatively valanced messages are likely to produce more negative tweets over time, while accounting for these historic events.

## 4. Results

### 4.1. Cross-Sectional Analyses of Tweet Sentiment Score

#### 4.1.1. Descriptive Statistics

The sample consists of 539,558 tweets containing gender and/or Asian slurs. The average sentiment was −1.02 (1.56 standard deviation) and the average text length was 123 (55 standard deviation). The full descriptive statistics for the cross-sectional dataset can be seen in Table 3.

#### 4.1.2. Examples of Tweets

Next, we highlight a few of the troublesome tweets from the original social media platform, paraphrased and deidentified.

##### Gender

In one highly retweeted example below, a user comments on how women can attack and be competitive with one another, writing that she is not their “sister” and they should “watch out.” In the second example, an individual uses multiple curses to attack a woman, claiming she will never forgive them for something they did:

B*tches are always throwing shade and trying to skate in there. I am not your sister b*tch watch out.

You’re a f*cking b*tch lol. F*ck you sl*t I’ll never forgive you for what you did to me lol f*ck off budak flat URL.

##### Asians

Of the many tweets in our data that exhibit hateful messages toward Asians, the one below invokes highly negative stereotypes, and it extends the abuse by calling on its victim to commit suicide:

I hope you die of cancer, stupid sewage Ch*nk r*t. K*ll yourself you d*ck sucking wh*re.

#### 4.1.3. Regression of Retweets

From our multivariate analysis results, shown in Table 4, we see that tweet sentiment is significantly and negatively related to logged retweets in Model 1. In Model 2, the control variables, number of friends and number of likes are positive and significant, whereas logged followers and tweet length have a negative and significant relationship with logged retweets. In our last model (Model 3), we add one event variable indicating if a tweet was created after COVID-19 was declared a pandemic. Including all key variables in Model 3, we find an improvement in comparison to both models 1 and 2, with the lowest Akaike Information Criterion (AIC) and Bayesian Information Criterion (BIC) values, and the highest adjusted R2. We find that the variable, logged friends, continues to be significant and positively related to logged retweets. Additionally, logged followers and tweet length are significant and negative. In contrast to Model 2, logged likes are now significant and negatively associated with logged retweets. Last, tweets posted after COVID-19 was declared a pandemic are significantly and negatively related to logged retweets. Overall, we find support for our first hypothesis: more negative tweets are associated with increases in numbers of retweets.

### 4.2. Longitudinal Analyses

#### 4.2.1. Descriptive Statistics

The final sample for the longitudinal dataset includes 248,497 tweets from 391 distinct users. As shown in Table 5, the average number of retweets was 6220 (with a median of 72), and the average number of likes was 1617 (with a median of 0), with an average logged retweet count of 4.29, and an average logged like count of 0.99. The average sentiment score was −0.11, with a standard deviation of 1.38. The median number of friends was 640, and the median number of followers was 543.

#### 4.2.2. Longitudinal Regression of Number of Retweets

We see that tweet sentiment is significantly and negatively related to logged retweets in a lagged Model 1, as hypothesized (see Table 6). Furthermore, the coefficient for logged retweets containing racial slurs, as compared to that for messages containing gender slurs, is significantly more negative (Model 1). One of the control variables in Model 2, logged followers, is positive and significant, whereas logged friends is insignificant. The control variable, “likes,” is negative and significant, and a positive, significant relationship exists between tweet length and logged retweets.

In Model 3, we include variables that indicate if a tweet was created after COVID-19 was declared a pandemic, and if a tweet was posted after Floyd’s murder. We find that tweet sentiment is still consistent in its significant, negative relationship to subsequent logged retweets. Tweets containing racial slurs, in comparison to those with gender slurs, remain significantly more positive in the model. In addition, logged likes have a significant, negative association with logged retweets, and tweet length is significant and positive. In contrast to Model 2, the variable, logged friends, has a significant and positive relationship to logged retweets. Last, tweets created after COVID-19, in comparison to those created prior, are significantly and negatively associated with logged retweets, whereas posts created after George Floyd’s death have a significant and positive relationship with logged retweets. Including all key variables in Model 3, we note an improvement over earlier models, with the lowest AIC and BIC values, and the highest adjusted R2.

#### 4.2.3. Longitudinal Regression of Tweet Sentiment

Finally, we investigate factors associated with tweet sentiment in a lagged model. From our multivariate analysis results, shown in Table 7, we see that the variable, logged retweets, is significantly and negatively related to later tweet sentiment in Model 1. Furthermore, the sentiment of tweets containing racial slurs is significantly more positive than that for tweets with gender slurs, (Model 1). The control variables in Model 2, logged followers and tweet length, are negative and significant, whereas logged likes are positive and significant, and logged friends is nonsignificant. In our final model, Model 3, we include variables that indicate if a tweet was created after COVID-19 was declared a pandemic, and if a tweet was posted after Floyd’s murder. In this final model, we see that the variable, logged retweets, continues to be significant and negatively related to subsequent tweet sentiment. Logged likes remain significant and positively associated with tweet sentiment, tweets containing racial slurs, compared to gender slurs, remain significantly more positive, and tweet length is significant and negative. Finally, both the COVID-19 and Floyd variables are significantly and negatively associated with tweet sentiment, as hypothesized. Notably, including all key variables in Model 3 represents an improvement over earlier models, with the lowest AIC and BIC values, and the highest adjusted R2.

**Figure 1:**
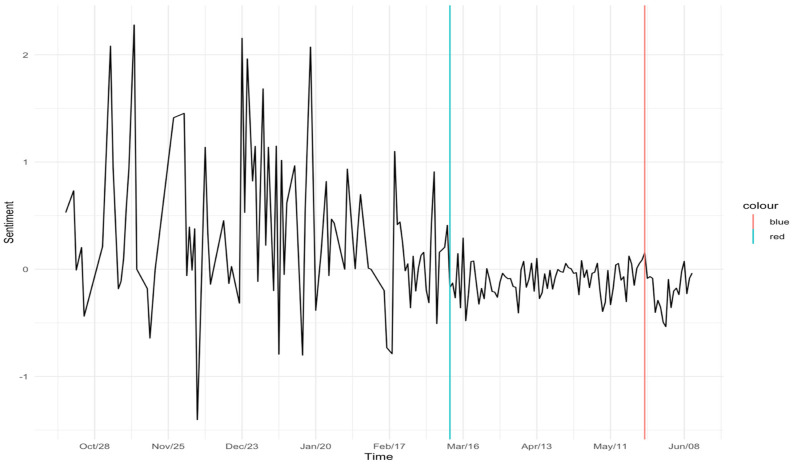
Average Sentiment over Time for Gender Keyword Users, with indicators for the times of the onset of COVID-19 (blue line) and Floyd’s Death (red line).

**Figure 2:**
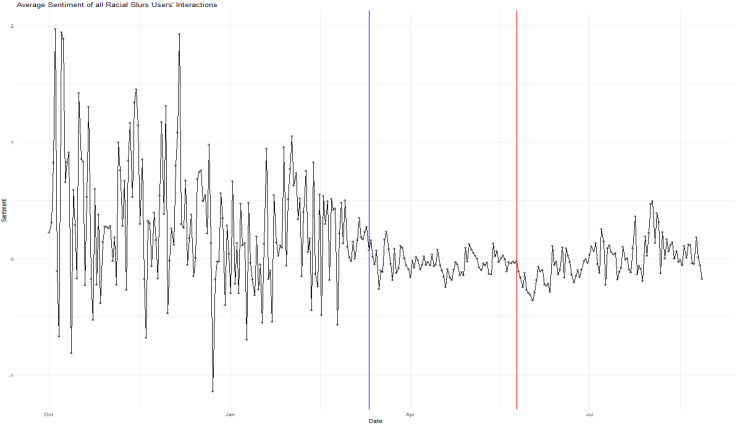
Average Sentiment over Time for Asian Keyword Users, with indicators for the dates of the onset of COVID-19 (blue line) and Floyd’s Death (red line).

The deleterious effects of the events of COVID-19 and Floyd on tweet sentiment can be seen visually in Figure 1 and Figure 2, presented separately for data based on users of gender and Asian keywords, respectively. These figures chart the average sentiment scores of users’ tweets beginning in October 2019 and ending in July of 2020. The blue horizontal line represents the formal onset of COVID-19, whereas the red line indicates the date of Floyd’s murder. In both cases, tweet sentiment is more negative following these two key external crises.

### 4.3. Robustness Checks

We conducted extensive robustness checks to test the quality of our results. First, we examined multicollinearity, finding that the variance inflation factors remained below 3 for all models. We also conducted an ordered logistic regression, finding comparable AIC values and no significant improvement in the models compared to the OLS regressions. Moreover, we estimated our models separately for the gender and Asian samples; conclusions regarding our main hypotheses did not differ, although the effect of COVID-19 in the gender cross-sectional analysis was nonsignificant. In addition, we controlled for the possible presence of “bots” in analyses not shown here, and found our conclusions remained unchanged.

Previous studies report that language detection systems used to identify hate speech can be subject to racial misclassification, with words such as “b*tch” incorrectly coded as abusive language [45]. To control for the possibility of misclassification, we excluded from our query search the term “b*tch”. We found no meaningful differences in significance level regarding our variables of interest, nor in coefficient sizes or signs, in the revised analyses. The adjusted R2 of the reduced model decreased significantly, however, so we proceeded with the original dataset.

Last, we reanalyzed our models without the “Asian” term, given that the word could be used in relatively neutral tweets. While the loss of tweets with the word “Asian” lowered the model fit substantially, it did not alter the key results, which is likely due to the frequent derogatory use of “Asian” in messages post-COVID-19.

### 4.4. Dispersion Patterns of Negative Tweets

**Figure 3:**
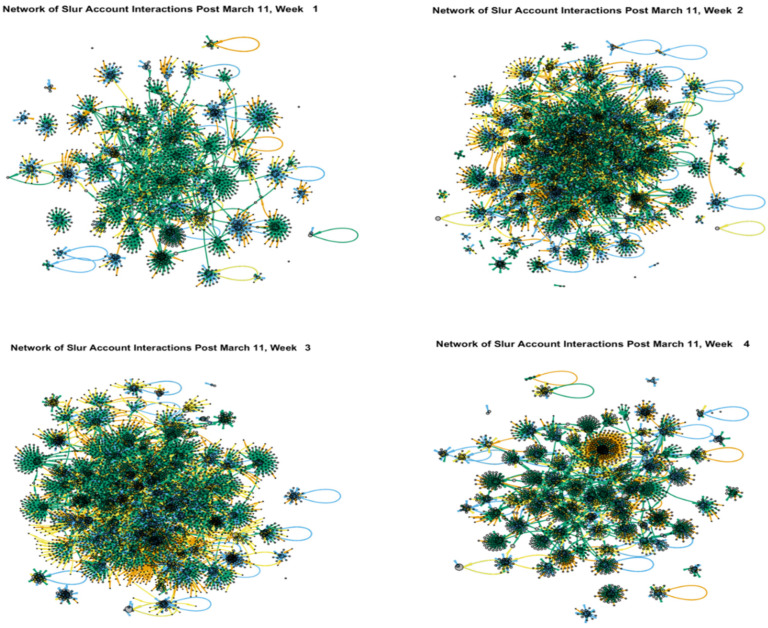
Networks of Sequential Twitter Interactions Using the Keyword “b*tch” during the 4 Weeks That Follow March 11.

**Figure 4:**
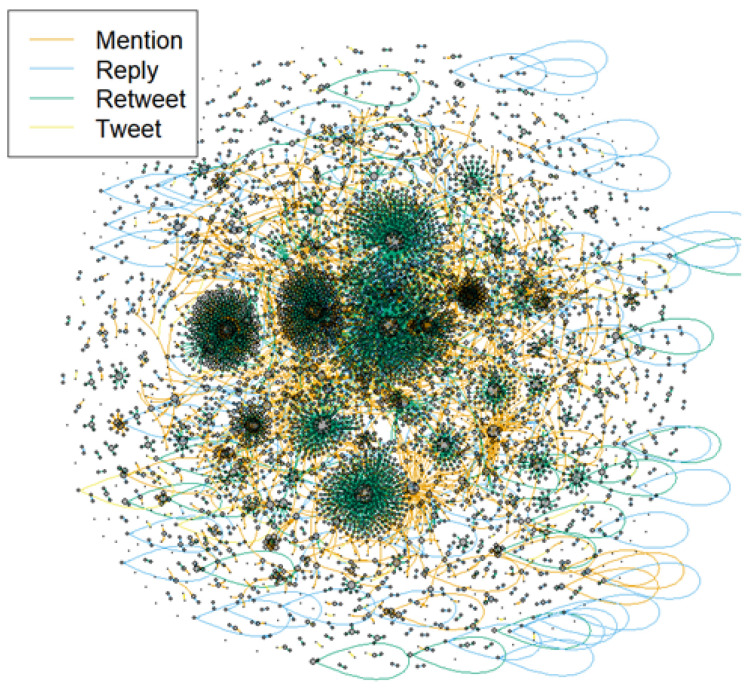
Network of Twitter Interactions with Highly Negative Asian, Racial Slurs from January 1 to June 30.

Online communication of negative tweets can range far beyond the original post, as seen in Figure 3 and Figure 4. Figure 3 illustrates the complex online interactions that developed on Twitter over four weeks in March 2020, all using the keyword, “b*tch”. Here, we see multiple pockets of retweets that became more extensive in the second and third week and then were less concentrated by the fourth week. Figure 4 depicts the detailed engagement patterns developing out of race-based slurs, and differentiates between patterns of mentions, replies, retweets and tweets. The dark areas in the center of the graph represent the high levels of retweets (indicated by the darkest lines) of messages containing Asian slurs. Overall, these examples illustrate the vast reach of offensive messages within online spaces.

## 5. Discussion

Internet communication provides a potential audience for anyone with thoughts they want to share. The rapid dissemination of commentary in an unregulated market applies to what is true or false, angry or measured, supportive or hateful, with the degree of dispersion facilitated by followers, routine readers, and social networks sharing information. Our results contribute to the literature regarding social processes involved in the spread of bullying on social media. First, we find that one reason individuals may post offensive material is because these tweets can garner greater attention than less negative posts. As hypothesized, our results show that negative sentiment in messages containing either gender or Asian slurs is associated with more retweets. Second, our findings also suggest that perpetrators successfully gain online notice by posting material that reflects predictably demeaning stereotypes, with tweets containing feminine gender and Asian slurs. In addition, our findings reveal a vicious cycle in which more negative tweets receive more retweets, and more retweets are associated with a rise in negativity of tweets. Finally, we find that the negative sentiment of tweets with such slurs increased following the onset of COVID-19 as well as after the killing of George Floyd, indicating the role of external shocks in exacerbating cyberbullying.

Overall, we find that more popular tweets (measured by number of retweets) are more negative in sentiment, a pattern that was clear in both cross-sectional and longitudinal analyses. The former provides evidence of negative sentiment being linked to higher levels of retweets at the time the message was scraped. The latter analyses demonstrate that more derogatory posts further accumulated retweets over the following days, with users who posted content with higher numbers of retweets also distributing more negative messages. Our figures of Twitter interactions, furthermore, illustrate the extensive dispersion of these offensive, popular messages within online spaces, a process that likely contributes to the virtual standing of a user who posts such a tweet.

Trends for the effects of numbers of likes, followers, and friends were mixed in our analyses and suggest that not all forms of social media engagement respond to negative posts. Likes, for example, were more frequent for upbeat messages. Reacting to a tweet with a ‘like’ expresses appreciation for message content, and therefore more apt to be linked to a positive post. But expressing a ‘like’ for a tweet does not necessarily increase its distribution. Only retweets directly disperse the tweet to new sets of viewers, thereby widening the circle of targets and amplifying awareness of the post.

Our results hold implications for theory by demonstrating that the group processes that characterize in-person encounters also emerge from virtual group interaction. People who post particularly negative, inflammatory messages are likely to see their post retweeted, which could be one way to boost their stature online and enhance their standing among fellow social media users. For people whose goal is to build readership, “going negative” works with tweets as well as with other media [29]. Our results align with findings from studies of aggression among students, where strategic uses of bullying boost adolescents’ friendship status both in person and online [25]. Our study implies that similar instrumental processes evolve within social media environments.

The tweets in our sample reflect detrimental, gender stereotypes. Some messages that contained typical, feminine curse words, for example, imply that women should be consistently sweet, nice, and chaste, while refraining from “rocking the boat” or acting in ways that could be considered “b*tchy” or sexually active. Moreover, according to one Twitter user in our sample (paraphrased): “When a woman is hated/harassed, her gender becomes an active player. Words like ‘sl*t’ or ‘b*tch’ focus on female sexuality, or being too feminine, or not being feminine enough, are always part of it”. Within this post, the user recognizes the damaging usage of gendered slurs and emphasizes how femininity and sexuality are weaponized against women.

Tweets also express hostility toward Asians, invoking negative stereotypes and devaluing or mocking the person. For example, racially charged language surrounded the COVID-19 pandemic, with tweets reinforcing adverse Asian sentiment through direct links, as in “kung flu,” “Asian virus”, and “ch*nk flu”, or by using a context-specific reference for the slur, “ch*nk”. Tweets that attacked Asians reflected xenophobic stereotypes suggesting that the actions of Asians harm society, and that Asian people deserve insults [46]. Similar processes appear to target Black individuals with the use of stereotyped slurs [47]. The repetition of abuse, and the concrete connection with negative, external events, offer clear signals to users who identify with the same “in-group” and choose to depersonalize and stigmatize members of marginalized groups in these digital spaces. These findings provide further evidence that the content of stereotypes is neither random nor capricious, but that it instead reflects a group’s disadvantaged social position [48].

### 5.1. Spread of Cyberbullying

Our findings highlight the preponderance of abusive messages on this form of social media. In the process of data collection, we located over one million (1,055,328) tweets that contained at least one of the keyword slurs. Through the simple action of retweeting, individuals can indicate support for an original, damaging tweet and spread its aggressive content to new networks of users. As negative content appears on an individual’s timeline, thus, the low cost of retweeting an abusive message likely contributes to the spread of this problematic content. These results relate to the way low costs can escalate aggressive, reciprocal responses in multiple contexts [49].

Our work supports extensive evidence from previous studies focusing on sexist and/or racist messages on social media [2,16,19,47]. According to Sobieraj [50], aggressive posts aim to silence public women, especially those from minority groups, and dissuade them from involvement in the public sphere. One outcome of bullying is that minority women victims can be compelled to withdraw from online activity, while engaging in costly, emotional labor to manage reactions to attacks [51].

Messages in our dataset that target women and Asians often fail to remain as isolated instances of communication between a pair of users. Rather, they can spread far beyond the initial post to extend to many others in the form of retweets, and in certain cases, reach surprisingly inflated numbers of users. The most damaging tweets, typically consisting of multiple curse words or highly negative phrases such as “kill yourself”, are especially prone to online spread. One of the most problematic aspects of digital bullying is the harm caused to victims by its public, widespread dissemination. Damage is unlikely to be limited to the original victim, furthermore. Tweets that receive more attention generate greater emotional contagion [52], suggesting that reposted tweets with gender and race slurs extend destructive emotions to additional users. Therefore, more research is needed to investigate ways to mitigate the spread of cyberbullying, especially given that it is possible to design policies to reduce hostility and abuse on social media [53,54].

### 5.2. Effects of COVID-19 and Floyd

Last, our analyses provide new insight into tweet sentiment and the ‘negativity process’ that social media can exacerbate. Since our data collection straddled both the WHO’s declaration of a pandemic and the death of George Floyd, we were able to assess how bullying aimed at two often targeted groups—Asians and women—reflected pre- versus post-event differences. Anti-Asian sentiment had been primed by news accounts and presidential pronouncements about the virus. Both the uncertainty and threat posed by the pandemic prompted adoption of the ‘scapegoating’ rhetoric to stigmatize Asians, and not surprisingly, the negativity of anti-Asian and feminine gender tweets increased after the WHO’s announcement that COVID-19 was a global pandemic. The societal unrest and distress following George Floyd’s demise also likely triggered cyberbullying, which produced more tweet negativity in our data following the tragedy. Not unlike previous research on political actions [55], our findings demonstrate that public events can significantly shift the emotional content of social media communication, and in our case, for the worse.

Although the current study has several strengths, it also has limitations. For example, Twitter users have been younger, more ethnically diverse, and more urban than the U.S. population [56], and as a result, our analyses do not reflect a broad swath of society. Our dataset also does not represent a random sample of all users who employ race and gender slurs on Twitter, and our findings cannot be generalized to the population of all such individuals. The true intent of tweets is difficult to measure, and some of our search terms can have multiple meanings. In some instances, the slur words we investigated are “reclaimed” to be used in a more positive sense that can be challenging to capture with a sentiment classifier, potentially introducing racial biases in measurement [45]. Sarcasm also is notoriously difficult to interpret with the use of automated systems. Although our customized sentiment classifier performed quite well when tested, and it was refined repeatedly to better handle such challenges, some degree of misinterpretation is inevitable. No one is completely immune to digital attacks, moreover, and research is needed to extend similar lines of inquiry into the social media treatment of other underrepresented and disadvantaged groups in our society, as well as those in more privileged positions. Finally, our investigation relies on a limited range of Twitter messages, and we do not know how these types of patterns are evolving in the new online messaging platform, X, obtained by Musk.

### 5.3. Implications for Practice

These findings have practical implications for professionals, such as therapists, educators, and social workers, who regularly deal with vulnerable, race and gender populations in society. In creating interventions to address the public health repercussions of cyberbullying for adults as well as adolescents [57,58], researchers and policy makers must consider how to integrate them into users’ daily lives. Professional awareness of the challenges documented herein, combined with the detrimental mental consequences for victims, underscore the importance of providing coping tools to those lacking social support or other resources necessary to handle these forms of hostility. Information about the nature of online bullying and its association with gender and race/ethnicity stereotypes could help raise awareness of this shared experience as a foundation for greater solidarity, coping mechanisms, and support when encountering such challenges. Additionally, understanding the spread and content of hostile messages raises awareness of the widespread exposure to cyberbullying and the perverse set of social norms that reward the worst offenders with shares that boost their visibility. The findings and methods described herein also could be used to aid in detecting certain cases of online bullying and aggression, potentially leading to new algorithms in the detection of users who may need additional support based on patterns of words and thematic content of hostile messages. Furthermore, if attention to negative tweets fuels online abuse, then one potential approach to reducing cyberbullying could be to diminish responses to these posts.

## 6. Conclusions

In conclusion, social media applications provide an all-too-common platform for internet bullying and abuse, with women and racial minorities frequent targets, and a ready podium that public crises only exacerbate. Why do individuals engage in such forms of belittlement? We maintain that they do so to move up the hierarchy of online visibility. Aggressors attempt to attain this goal by spinning messages that are seen by others, that are easily disseminated to novel networks via retweets, and that endorse normative, negative stereotypes and prejudice. Derogatory content in social media is not idiosyncratic in nature, but rather strategic and akin to face-to-face social interaction. One key difference is that cyberbullying has the potential to quickly reach a much more sizeable audience by expanding deeply into virtual space, with the possibility of extensive harm. Further work is needed to study the intricate, social processes inherent in the evolution of this pernicious, societal problem.

## Figures and Tables

**Table 1:** Frequencies for Keywords Searched, Cross-Sectional Data.

Keyword	Frequency
B*tch	233,024
Sl*t	211
Wh*re	179
C*nt	119
Asian	163,029
Ch*nk	16,554
Hong Kong Flu	12,898
Chinese Virus	64,071
Kung Flu	70,614

**Table 2:** Frequencies for Keywords Searched, Longitudinal Data.

Keyword	Frequency
B*tch	956
Sl*t	16
Wh*re	7
C*nt	4
Asian	985
Ch*nk	18
Hong Kong Flu	12
Chinese Virus	150
Kung Flu	40

**Table 3:** Descriptive Statistics for the Cross-Sectional Dataset.

	Mean (Standard Deviation)	Median	(Min, Max)
Retweets	3442 (12,142)	71	(0, 1,046,053)
Likes	6803 (24,030)	0	(0, 1,107,651)
Friends	1811 (6621)	454	(0, 664,607)
Followers	4801 (165,335)	383	(0, 57,397,274)
Text Length	123 (55)	139	(3, 368)
Sentiment	−1.02 (1.56)	−1.06	(−4, 4)
Logged Retweets	4.21 (3.65)	4.28	(0, 13.86)
Logged Likes	2.72 (4.12)	0	(0, 13.92)
Logged Friends	6.17 (1.58)	6.12	(0, 13.41)
Logged Followers	5.9 (1.98)	5.95	(0, 17.87)

**Table 4:** OLS Regressions, Regressing Retweets, Cross-Sectional Data.

Variable	Model 1	Model 2	Model 3
Intercept	4.008 ***	3.233 ***	3.392 ***
Sentiment Score	−0.127 ***	−0.134 ***	−0.132 ***
Logged Likes		0.011 ***	−0.012 ***
Logged Friends		0.321 ***	0.320 ***
Logged Followers		−0.157 ***	−0.156 ***
Text Length		−0.004 ***	−0.004 ***
After COVID			0.099 ***
R^2^	0.0029	0.0125	0.0125
Adj R^2^	0.0029	0.0125	0.0125
AIC	3,377,095	3,367,961	3,367,941
BIC	3,377,129	3,368,040	3,368,032

*** *p* < 0.001.

**Table 5:** Descriptive Statistics—the Longitudinal Dataset.

	Mean (Standard Deviation)	Median	(Min, Max)
Retweets	6220 (24,431)	72	(0, 993,003)
Likes	1617 (11,355)	0	(0, 373,379)
Friends	3142 (7730)	640	(0, 69,331)
Followers	3986 (11,541)	543	(0, 87,938)
Text Length	105 (51)	119	(0, 308)
Sentiment	−0.11 (1.38)	0	(−4, 4)
Logged Retweets	4.29 (3.77)	4.29	(0, 13.81)
Logged Likes	0.99 (2.59)	0	(0, 12.83)

**Table 6:** Fixed-User Models, Longitudinal Data, Regressing Retweets.

	Null Model	Model 2	Model 3
(Intercept)	6.831 ***	18.047 ***	19.313 ***
Sentiment Score	−0.102 ***	−0.070 ***	−0.065 ***
Dataset: Race	−4.757 ***	−20.571 ***	−19.537 ***
Logged Likes		−1.405 ***	−1.401 ***
Logged Friends		0.045	0.093 ***
Logged Followers		0.549 ***	0.177 ***
Tweet Length		0.011 ***	0.011 ***
After COVID-19			−0.189 **
After Floyd’s Murder			0.368 ***
Mult. R2	0.4126	0.4567	0.4584
Adj. R2	0.4117	0.4558	0.4575
AIC	1,233,105	1,213,758	1,212,983
BIC	1,237,201	1,217,895	1,217,141

** *p* < 0.01; *** *p* < 0.001.

**Table 7:** Fixed-User Models, Longitudinal Data, Regressing Sentiment Score.

	Null Model	Model 2	Model 3
(Intercept)	0.177 *	0.701 ***	0.427 **
Logged Retweets	−0.022 ***	−0.016 ***	−0.015 ***
Dataset: Race	0.860 ***	1.449 ***	1.126 ***
Logged Likes		0.013 *	0.013 *
Logged Friends		0.012	−0.004
Logged Followers		−0.150 ***	−0.029
Tweet Length		−0.002 ***	−0.002 ***
After COVID-19			−0.106 ***
After Floyd’s Murder			−0.120 ***
Mult. R2	0.0449	0.0490	0.0504
Adj. R2	0.0433	0.0475	0.0489
AIC	853,723	852,641.8	852,277.1
BIC	857,819	856,779.6	856,435.7

* *p* < 0.05; ** *p* < 0.01; *** *p* < 0.001.

## Data Availability

The Twitter dataset was collected via the Twitter Application Programmer Interface and cannot be shared, because it represents third-party information that was restricted by Twitter’s terms of service at the time of data collection. However, we provide details of the search parameters used to construct this dataset (see Table 1 and Table 2).
